# Why Do I Need Sleep? Exploring Children’s Views on Sleep and Its Importance

**DOI:** 10.3390/healthcare14050611

**Published:** 2026-02-28

**Authors:** Nandini Adusumilli, Kate O’Halloran, Xóté Tadhg Ó Séaghdha, Yasmeen Al Saud, Dagmara Dimitriou

**Affiliations:** 1 Sleep Education and Research Laboratory, UCL Institute of Education, London WC1H 0AA, UK; nandini.adusumilli.21@ucl.ac.uk (N.A.);; 2 ‘Better than Cure’—The International Preventive Medicine Agency, Via Lucano, 6982 Agno, Switzerland; 3 Imperial College Healthcare NHS Trust, London W2 1NY, UK

**Keywords:** sleep problems, sleep practices, children’s sleep, school performance

## Abstract

**Background/Objectives**: Sleep plays a crucial role in children’s cognitive, emotional, and physical development. Although sleep practices and perceptions are shaped significantly by cultural and familial contexts, most sleep recommendations are developed by Western countries. This qualitative study explores primary school children’s perceptions of sleep, examining how cultural contexts, family environments, and technology influence sleep practices. **Methods**: Two cross studies were conducted: Study 1, in India, involved 15 children aged 8–12 years, and Study 2, in the UK, involved 12 children aged 8–10 years. Semi-structured group interviews and thematic analysis were used. **Results**: Both studies revealed common themes, including perceived sleep benefits, consequences of poor sleep, factors affecting sleep quality, and the role of technology. Study 1 showed that Indian children identified clear benefits of sleep, such as physical and emotional well-being, while highlighting significant barriers, including late bedtime routines, stress related to academic performance, and extensive use of social media and digital media devices. Cultural and religious practices were commonly mentioned as sleep aids. Study 2’s results from the UK revealed similar recognition of sleep benefits, notably recovery and growth. UK children emphasised environmental barriers such as noise pollution, sibling disturbances, and uncomfortable sleeping conditions. Technology usage was acknowledged as both a barrier and an occasional aid, with stricter parental controls on bedtime and device usage. **Conclusions**: This research highlights the importance of culturally sensitive sleep education programmes and recommendations to enhance children’s sleep health globally.

## 1. Introduction

Sleep is a vital determinant of children’s well-being [1,2]. Recent studies confirm the growing concern of sleep deprivation in young children [3,4], with increasing screen time and exposure to digital devices further compounding sleep disturbances, particularly when used late in the evening [5]. Sleep is an inherently complex phenomenon, influenced by intricate interactions across genetic [6,7], physiological [8], psychological [9] and cultural domains [10]. Consequently, examining sleep in isolation fails to capture its multifaceted nature [11,12]. Investigating sleep practices and perceptions within diverse cultural and environmental contexts provides valuable insights into how individuals understand and experience sleep [13,14]. Such research is essential for developing holistic, culturally informed strategies to promote sleep health on a global scale [15,16,17].

Despite the growing body of sleep research, sleep is often assessed from adult or clinical viewpoints, overlooking children’s subjective experiences [18,19,20]. Recent participatory studies have shed light on how children perceive and articulate their sleep needs and challenges. For example, Landwehr [21] employed the photovoice method to investigate primary school children’s perspectives on healthy sleep. By engaging children as active participants, the research revealed their nuanced understanding of factors impacting sleep, such as bedtime routines, environmental disturbances, and family dynamics. Contradictions between children’s perceptions of sleep and their lived realities were evident, with participants identifying inconsistencies in bedtime practices as a significant barrier to achieving restorative sleep, suggesting the need for family-centred interventions to support consistent routines.

Additionally, increasing screen time and exposure to digital devices further compounded sleep disturbances, particularly when used late in the evening [5]. However, there remains a limited understanding of children’s perspectives on sleep, especially in non-Western cultural contexts such as India [22,23,24,25]. The increasing use of technology among children worldwide, including screen time and digital media [26,27], is increasingly influencing sleep patterns and routines [28], yet this area remains underexplored in diverse cultural settings, especially the inclusion of children’s perspectives.

A study by Stickland et al. [29], which was carried out in hospital settings, showed that changing the sleeping environment can have a profound impact on sleep. For example, children identified environmental disruptions such as noise, light, and unfamiliar settings as key barriers to a good night’s sleep. The children’s reports of discomfort and lack of control highlighted the importance of creating sleep-conducive environments, even in challenging contexts such as hospitals. Harvey et al. [30] further explored children’s subjective experiences of sleep, emphasising their ability to articulate concerns about sleep onset and quality of sleep. These findings suggest that children’s voices are an underutilised resource in understanding sleep barriers and addressing sleep issues.

### 1.1. Cultural and Environmental Influences

The American Academy of Paediatrics and the World Health Organization have provided recommendations for the number of sleep hours, as well as for media use in relation to age [31,32]. However, recent research suggests that sleep patterns are heavily shaped by cultural values, beliefs, and practices. In their systematic review, Jeon et al. [33] highlighted that sleep patterns can be influenced by a variety of factors, including the environment, economic conditions, parenting styles, screen time, and sleeping arrangements. Factors that influence sleep in one country may not have the same impact in another, highlighting how cultural differences play a role in shaping sleep patterns.

For example, a study by Takahashi et al. [34] comparing preschool-aged children in Japan and China found notable differences in their sleep patterns, including bedtime, wake-up time, and total sleep duration. Chinese preschoolers experienced more nighttime awakenings than their Japanese counterparts, which may be linked to higher rates of sleep-disordered breathing caused by greater exposure to air pollution in China compared to Japan. This highlights the need for sleep guidelines and interventions to take cultural and environmental differences into account, especially when making age-specific recommendations for healthier sleep habits across different regions.

Similarly, Ramar et al. [22], examining Indian children, highlighted how environmental factors such as later sunsets in India directly impacted the sleep duration of school-aged children. Children reported staying up late at night, despite having unchanged wake-up times due to strict morning routines, preventing them from making up for lost sleep. This reduction in sleep time was found to significantly hinder academic performance by decreasing the time spent on homework and study-related activities. Lack of nighttime sleep was compensated for by taking more daytime naps and engaging in indoor leisure activities. Emotional dysregulation, another key finding, was tied to lack of sleep, further complicating students’ abilities to engage in their learning in school and social environments. It emphasised the influence of cultural practices, such as family evening routines and social expectations, on children’s sleep patterns.

Building on this, Twenge et al. [26] conducted a comparative study of sleep hygiene and quality in Indian and Saudi Arabian cultural contexts, the research analysed data from 185 participants, including 95 from India. The study found that Indian participants often followed traditional sleep practices, such as drinking warm milk or herbal teas before bed. Furthermore, family structures in joint households and social obligations played a significant role in shaping sleep schedules, sometimes resulting in irregular sleep patterns.

Children who have shorter sleep durations are at risk of facing a variety of challenges. These can include problems with both physical and mental health, difficulties with learning and memory, struggles to focus, hyperactivity, and lower academic performance [35,36]. Given the wide-ranging negative effects of sleep deprivation, it is crucial to prioritise and encourage healthy sleep habits in children to support their overall well-being and development.

### 1.2. The Current Study

This qualitative study aims to provide a foundation for future large-scale, cross-cultural research to better understand how cultural norms, environmental factors, and technology collectively shape children’s sleep behaviours. This work will contribute to a more inclusive understanding of sleep’s role in child development globally by providing insights from school-aged children in India and the UK regarding their sleep habits and perspectives on sleep.

This qualitative study aims to gather insights school-aged children in India and the UK regarding their sleep practices and perspectives on sleep. By addressing this gap, the study seeks to provide a foundation for future large-scale, cross-cultural research to better understand how cultural norms, environmental factors, and technology collectively shape children’s sleep behaviours. This work will contribute to a more inclusive understanding of sleep’s role in child development globally.

## 2. Materials and Methods

The study was carried out in two countries, namely India and the UK.

### 2.1. Participants

Children in India: A total of 15 primary school students (male = 7, female = 8) aged 8–12 years old. Participants were recruited from a private school in Gadag, Karnataka. Convenience sampling was utilised with the help of the schoolteachers. Children in the UK: A total of 12 primary school students (male = 5, female = 7) aged 8–10 years old. Participants were recruited from a voluntary-aided Roman Catholic Primary School in London and were of mixed socio-economic backgrounds. The sample size was determined by the qualitative design of the study and guided by the principle of data saturation, whereby data collection continues until no new themes emerge. According to [37], three to six groups are sufficient to capture 90% of themes, including the most prevalent themes, in a homogenous study using a semi-structured discussion guide. The study, therefore, involved three groups for interviews. Group interviews offer a valuable and versatile platform that gives the participants an opportunity to interact and reflect on the topic.

Convenience sampling was used to recruit participants through schools. The sample size was determined by the qualitative design of the study and guided by the principle of data saturation, whereby data collection continues until no new themes emerge. Previous qualitative research indicates that relatively small samples can be sufficient to capture key themes in homogeneous groups of children using focus group interviews [37,38]. In the present study, responses began to repeat across focus groups, indicating that thematic saturation had been reached within the sampled contexts.

### 2.2. Procedure

Group interviews took place face-to-face in an intervention room in the school, which was reserved for part of the day. Students were allocated to groups of three, with five children in each group. Children were orally informed about the study prior to the session, and the session began with an ice-breaker round to make the children comfortable. Interviews were recorded on an electronic device. Questions included “What helps you to sleep at night?” “What makes it difficult for you to fall asleep at night?” and “What kinds of electronic devices do you have access to in your bedroom?” The semi-structured nature of the interviews meant that some additional questions were asked to clarify the children’s responses and to facilitate them in building on prior responses. This was to ensure that the data collected was rich and insightful. To minimise interruption to learning time, the interviews lasted 40–45 min.

### 2.3. Thematic Analysis

All written responses in each focus group were copied and pasted into a Word document verbatim. The data from the focus groups were then analysed using qualitative thematic analysis. Thematic analysis followed the six stages of [38]’s approach of reading and re-reading the transcripts to build familiarity with the data before a line-by-line analysis of the transcript. Firstly, the data was divided into the form of questions and relevant answers. After this, initial codes were generated by categorising repetitive responses by identifying similarities in the data. Then, similar or related codes were grouped into potential categories using a thematic map from which the final themes were identified, reviewed, and named. Final themes and subthemes were then reviewed again and agreed upon by two researchers (NA, KO). Lastly, results were presented in a comparative format by discussing in detail each theme that was generated and the codes under it, finding similarities and differences within and between focus groups, and adding relevant participant quotes to support the results.

The exploratory nature allowed for the collection of deep and rich insights into student perceptions about sleep, which is often difficult to capture using quantitative research, which focuses on numerical data points [39]. Thematic analysis was used to allow the identification of important patterns within the data and subsequent detailed interpretation of children’s sleep perceptions and bedtime technology use. Group interviews were chosen to explore children’s sleep perceptions, the facilitators and barriers to their sleep and their technology use around bedtime. Group interviews are particularly useful for gaining insight into a target group’s experiences and perceptions regarding an idea, an issue, or a phenomenon.

### 2.4. Ethical Consideration

Ethical consideration was granted by University College London (UCL) under the Institute of Education (IOE), along with the local Indian ministry, with the help of Elk. Health Research Centre. Information sheets and consent forms were sent to parents, and children who returned signed consent forms were recruited for the study. Data Protection Number: Z6364106/2025/01/82. The headteachers of schools were informed of the study both verbally and in writing. They received an information sheet and a formal letter requesting permission to undertake the study with students in the school. Once permission was granted, dates were agreed for the group interviews to take place. Participants were reminded that their answers would be kept confidential; they did not have to answer any questions they did not want to; and that they could withdraw from the study at any time up until a given date. All participants were assigned a corresponding identification code to ensure the anonymity of the data.

## 3. Results

The results section is divided into two studies, namely Study 1, carried out in India, and Study 2, carried out in the UK.

### 3.1. Study 1: Children’s Focus Groups in India

**Figure 1:**
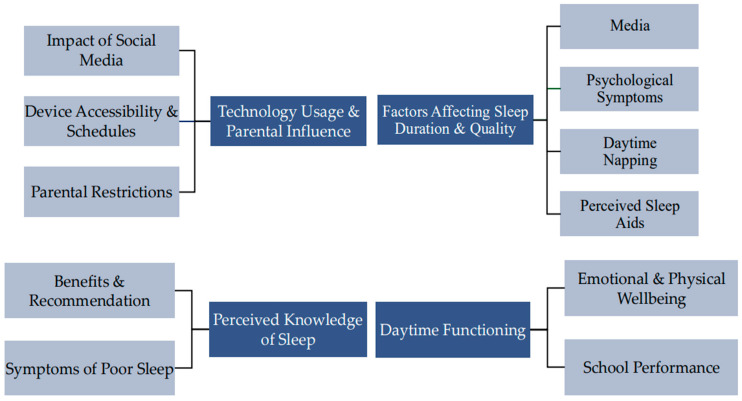
Four key themes and subthemes identified in Study 1.

The thematic analysis of the transcripts identified four key themes: children’s perceived understanding of sleep, factors influencing sleep duration and quality, daytime functioning, technology usage and parental influence. See Figure 1 for a diagram of themes and subthemes.

### 3.2. Theme One: Perceived Knowledge of Sleep

This theme explores children’s understanding of sleep based on perceptions of their factual knowledge. It is further divided into two sub-themes: the benefits of sleep, as recognised by the children, including the positive recommendations they wished to share, and the perceived consequences of sleep deprivation.

#### 3.2.1. Benefits and Recommendations

All the children unanimously acknowledged the importance of sleep for overall functioning and health.

For instance, Child 2 stated, *“We need to sleep to be ready for the next day,”* while Child 3 expanded on this by saying, *“Your mind relaxes when you sleep, helping us stay active. Our body and eyes feel refreshed.”*

Some children also shared their recommendations for healthy sleep habits. For example, Child 3 stated, *“Adults should sleep for 7–8 h; we need to sleep for 9–10 h, and babies should sleep for 10–12 h.”*

Adding to the discussion, Child 4 suggested, *“We need to sleep by 8 PM and wake up by 7 AM.”*

#### 3.2.2. Symptoms of Poor Sleep

Many children were eager and enthusiastic to share the negative effects of sleep deprivation, immediately highlighting serious health concerns.

For example, Child 6 stated, *“Lack of sleep can lead to diseases like heart problems, headaches, diabetes, and even dark circles under our eyes.”*

Similarly, Child 1 added, *“If we don’t sleep, we will develop dark circles and headaches.”*

### 3.3. Theme Two: Factors Affecting Sleep Duration and Quality

This theme examines the factors influencing children’s sleep quality from their own perspective. It is further divided into three sub-themes: psychological symptoms, which address their mental struggles related to sleep; media usage and difficulties with daytime napping; and helpful sleep aids, as perceived by the children.

#### 3.3.1. Psychological Symptoms

Some children identified mental health challenges as a barrier to their sleep, often citing academic stress and psychological struggles. For instance, Child 9 shared, *“Sometimes, even when I’m not using a device, I struggle to sleep because of my thoughts.”* Similarly, Child 1 stated, *“When I feel tension or stress about exams, I find it difficult to sleep.”* Expanding on the impact of school pressure, Child 5 shared, *“Sometimes schoolwork makes me feel sleepy, but when I try to sleep at night, I just can’t.”*

#### 3.3.2. Daytime Napping

Additionally, a few children mentioned that daytime napping negatively impacted their nighttime sleep, though it was not a widely discussed topic among the groups. For example, Child 4 stated, *“When I nap in the afternoon, I can’t sleep at night,”* while Child 6 added, *“If I rest for too many hours during the day, I struggle to sleep at night.”*

#### 3.3.3. Media

Media usage was widely recognised by children as a factor affecting sleep. For example, Child 2 stated, *“When we watch horror movies, I find it difficult to sleep.”* Child 3 agreed and added, *“When we skip meals and watch horror movies, it becomes even harder to sleep.”* Watching horror content appeared to be a common trend among many children as the discussion progressed. Child 13 shared, *“Even if I watch horror for just 20 min, it keeps me up late at night.”* This sparked a conversation within the focus groups about why horror is often watched at night despite its obvious negative effects. In response, Child 6 explained, *“It’s only fun to play horror games at night.”* Interestingly, one child shared a unique perspective, stating, *“Horror storybooks help me fall asleep.”*

#### 3.3.4. Perceived Sleep Aids

Children identified a variety of sleep aids they believed helped them fall asleep at night. These can be broadly categorised into: religious/cultural practices, reading books/listening to stories, diet, topical treatments and media.

A few children from different religious backgrounds mentioned that their religious texts helped them fall asleep. For example, Child 6 shared, *“I listen to the Hanuman Chalisa to fall asleep,”* referring to the Hindu devotional hymn known for warding off evil. Similarly, Child 8 stated, *“I read a religious book—the Quran—to fall asleep,”* referring to the central religious text of Islam.

Continuing the theme on sleep aids, storybooks and media emerged as a widely agreed-upon method for falling asleep, along with maintaining a proper diet to support better sleep quality. For example, Child 1 highlighted both factors, stating, *“Reading storybooks helps me sleep, and using my phone a lot also makes me sleepy.”* When considering diet, Child 3 mentioned, *“If I don’t eat dinner, I can’t sleep, and using extra pillows helps me fall asleep.”* Similarly, Child 2 added, *“A proper diet helps us sleep.”*

Lastly, Child 8 shared, *“I apply Zandu Balm to fall asleep.”* Zandu Balm is a popular remedy in India, commonly used for quick relief from respiratory issues such as nasal congestion and headaches. Its key ingredients include eucalyptus oil, turpentine oil, menthol, and camphor, which are generally known for their soothing and calming effects.

### 3.4. Theme Three: Daytime Functioning

This theme explores various aspects of efficient daytime functioning as perceived by children. It is divided into three sub-themes: emotional and physical well-being, based on children’s self-assessments, and school performance in relation to sleep duration and quality, where some children expressed differing opinions on the connection between sleep and daytime functioning.

#### 3.4.1. Emotional and Physical Well-Being

Almost all children generally agreed that their sleep directly impacted their emotional and physical well-being the following day. For example, Child 2 stated, *“I feel stressed at school the next day due to a lack of sleep.”* Similarly, Child 6, referring to sleeping late, shared, *“I feel angry and irritated, not just in school but throughout the day because I’m just so sleepy.”*

Expanding on this theme, nearly half of the children in the groups expressed a feeling of needing more sleep. For example, Child 4 shared, *“On weekends, even though I sleep until almost noon, I still feel like I need more sleep.”* Similarly, Child 3 remarked, *“I felt it today… despite sleeping early yesterday, I don’t know why.”*

#### 3.4.2. School Performance

While discussing school performance, most children strongly agreed that the quality of sleep the night before had a significant impact on their ability to perform well in school. Similarly, Child 5 shared, *“When I don’t use my phone on certain occasions and sleep early, I notice that I feel more refreshed at school the next day.”* Also, Child 1 stated, *“I feel stressed at school the next day due to a lack of sleep.”*

However, a minority of children disagreed that sleep had any connection to daytime functioning or school performance. Two children mentioned media as a factor influencing their energy levels. For instance, Child 10 stated, *“I don’t think that using devices or sleep has any impact on my school day. I feel the same; it doesn’t affect me in any way. Sleep has no connection with school.”* Similarly, Child 3 added, *“Using my phone makes no difference to me. In fact, things at school feel even more interesting.”*

### 3.5. Theme Four: Technology Usage and Parental Influence

This theme primarily explored the role of technology and its significant impact on children’s lives, as well as the influence of parents in shaping their ideologies and enforcing rules, from the children’s perspectives. It was thus divided into three subcategories: the role of social media and children’s usage, device availability at home, and parental restrictions and ideologies influencing technology use.

#### 3.5.1. Impact of Social Media

Nearly every child, despite their young age, mentioned being active on social media platforms such as Snapchat, Instagram, Facebook, and WhatsApp. Mindless scrolling on YouTube shorts seemed to be very common and was agreed upon by all. For example, Child 9 shared, *“I use Roblox—only sometimes if I get bored. Otherwise, I use YouTube, Facebook, Instagram, and WhatsApp.”* Similarly, Child 7 stated, *“I use WhatsApp, Instagram, Google Chrome, games, and Football 2024.”* When asked about the connection between technology and sleep, the children unanimously agreed that social media, video games, and television often kept them up very late at night. For example, Child 12 shared, *“Watching YouTube Shorts keeps me up, and I end up sleeping at 11 PM or 12 AM.”* Similarly, Child 5 from another group stated, *“I watch YouTube Shorts, so I end up sleeping between 11 PM to 12 AM; otherwise, I sleep between 10 to 11 PM.”*

#### 3.5.2. Device Accessibility and Schedules

The earliest common bedtime, based on the average across the focus groups, appeared to be 11 PM, but many children admitted to staying awake until 1–2 AM on school nights, indicating that late-night sleep has become a typical habit among them. For instance, Child 11 shared, *“Playing online games like Candy Crush and Jalebi keeps me up, and I end up sleeping at 12 AM.”* Meanwhile, Child 4 stated, *“Mobile phones don’t keep me up, but watching TV does, so I sleep between 10 to 11 PM every day.”*

This led the focus group discussion toward exploring where children use their devices at home. The responses varied widely. Most children admitted using devices in the same room where they sleep, especially closer to bedtime, even if they did not keep them with them throughout the night. Some justified this by saying they used devices for schoolwork, while others explained that their computers and phones were simply located in their bedrooms where they sleep with their parents. For example, Child 5 shared, *“At nighttime, when my mother is done using her phone, she gives it to me for five minutes, and then it’s kept beside the bed.”*

Finally, the children were asked to explain, from their perspective, why they chose to use devices only at bedtime or closer to bedtime. This led to almost all children expressing frustration over their hectic academic schedules, which included school, homework, dinner, and playtime, leaving them with only bedtime or late-night hours to use their devices. However, it was evident that most children’s form of relaxation involved less physical play and more digital media consumption, such as watching television, playing video games, and using social media to unwind. For example, Child 8 explained, *“We have to get ready for school in the morning. I get home around 4:30 PM, and by the time I finish my homework and eat dinner, it’s 8:00 PM. So, I use my devices until 10:30 PM and then go to sleep.”* Similarly, Child 4 shared, *“After school, I have to do my homework, and by the time I finish, it’s 8 PM. So, I use my phone before bedtime because the only other time I have is early in the morning before school, and I can’t wake up that early.”*

#### 3.5.3. Parental Restrictions

From the children’s perspective, parental restrictions appeared relatively lenient, especially regarding late bedtimes. However, they did not seem to resist the few rules their parents enforced, such as when they were allowed to have a phone or their designated sleep and wake-up schedules. This compliance also aligned with their tendency to unquestioningly adopt their parents’ beliefs and ideologies. However, it was quite apparent that parents did not fully understand how fatigued their child might be when enforcing sleep and wake-up schedules.

For instance, Child 6 mentioned, *“On Saturdays, I tend to sleep late because it’s the weekend, but my mother still wakes me up early on Sundays, so I feel tired.”* Similarly, Child 9 expressed, *“On Sundays, I have to wake up by 6:30 AM for karate class, despite having a bedtime of 11 PM, so I feel very tired.”* Regarding adherence to timed device usage due to parental restrictions, Child 9 stated, *“My mother lets me use her phone for an hour, and I like to go on Facebook, which leads me to sleep around 12 AM.”* However, the late bedtime largely went unmonitored, as was the case with most children. Lastly, to further support the idea of beliefs and ideologies being passed down, child 9 remarked, “It’s not so fun to use my phone in the mornings, and my mother says that using it in natural sunlight harms my eyes, but it’s okay to use devices under LED light at night.” Although not scientifically backed, the children were too young and unquestioningly trusted their parents.

### 3.6. Study 2: Children’s Focus Groups in the UK

**Figure 2:**
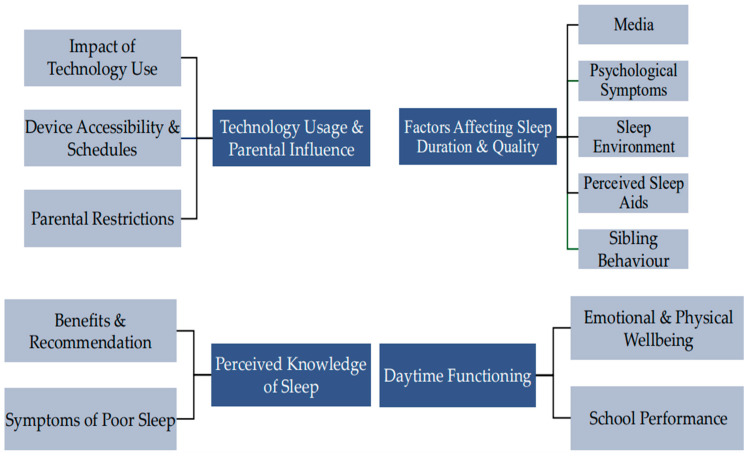
Four key themes and subthemes identified in Study 2.

This study was undertaken in a primary school in London. The study adopted a qualitative design, where year 4 and year 5 students engaged in semi-structured group interviews. Thematic analysis was used to allow the identification of patterns within the data and to subsequently gain an understanding of children’s knowledge of sleep, the facilitators and barriers to their sleep, and their technology use around bedtime, as perceived by the children. See Figure 2 for a diagram of themes and subthemes.

### 3.7. Theme 1: Perceived Knowledge of Sleep

This theme describes the perceived knowledge of sleep held by the participants. This theme has been further subcategorised into two sub-themes: benefits and recommendations, and symptoms of poor sleep.

#### 3.7.1. Benefits and Recommendations

When considering the benefits and recommendations, participants reported that sleep provides them with energy. Child 10 mentioned how they believed *“sleep gives us rest, and energy.”* Child 3 also said that *“you get energy from it.”* Other participants perceived recovery and growth to be a benefit of sleep. Child 11 expressed that *“sleep helps us recover from the day we’ve just had.”* Child 9 added, *“And if you’re sick, sleep helps you to recover. It makes you feel fresh.”* Child 3 commented that *“sleep helps you to grow.”*

#### 3.7.2. Symptoms of Poor Sleep

In relation to symptoms of poor sleep, participants frequently described uncomfortable physical symptoms. For example, Child 2 explained that when they do not sleep well, they *“get a headache”* and *“it’s painful.”* Along with headaches, Child 6 mentioned that *“you’d be very dizzy too”* without adequate sleep. Many participants also reported that poor sleep negatively impacted their performance in school. For instance, Child 2 said: *“I’m usually good in Maths. But the next day, when I’ve slept badly, I just feel tired so I can’t really be bothered to do it that much.”*

### 3.8. Theme 2: Factors Affecting Sleep Duration and Quality

This theme encompasses the factors that the children believe affect sleep duration and quality. This theme is divided into five sub-themes: psychological symptoms, media, sibling behaviour, sleep environment and perceived sleep aids.

#### 3.8.1. Psychological Symptoms

Children perceived psychological symptoms as being a barrier to sleep. Child 9 stated that it’s difficult to sleep *“when you’re scared of something.”* Child 12 added that it is also difficult to sleep *“when you’re too excited for the next day. Like when it’s Christmas.”* Excessive thinking was also described as a sleep barrier, as Child 10 stated: *“Well, really, I don’t sleep well when I think a lot.”* Child 9 described how they find it difficult to fall asleep when their thoughts lead to visions: *“Yeah visions! Like sometimes you see scary things when your eyes are closed before you go to sleep.”*

#### 3.8.2. Media

Children frequently described *media* as being a sleep barrier. Child 11 stated that when watching a movie, she did not feel like sleeping: *“when my mum comes in when I’m watching something and tells me to go to bed, I’m like I don’t want to go to bed, I’m mid movie!”* Child 3 said *“Youtube”* keeps them awake past their bedtime, and *also “Google because when I finish watching a movie and I see one of my favourite actors or actresses in it, I have to search them up and find out information about them.”* Child 12 thought that videogame playing affected their sleep *“very badly. It just makes me feel like I can’t go to bed, because I really want to finish the game, so I can’t really go to bed.”*

Technology was used by most participants and was commonly used in the time preceding bedtime. Technology use was commonly described as having a negative impact on sleep and seemed to delay the sleep onset of the children. Child 12 thought that videogame playing affected their sleep *“very badly. It just makes me feel like I can’t go to bed, because I really want to finish the game, so I can’t really go to bed.”* Child 11 stated that when watching a movie, they did not feel like sleeping: *“when my mum comes in when I’m watching something and tells me to go to bed, I’m like I don’t want to go to bed, I’m mid movie!”* Child 3 said *“Youtube” keeps them awake past their bedtime, and “Google, because when I finish watching a movie and I see one of my favourite actors or actresses in it, I have to search them up and find out information about them.”* On the other hand, video and TV watching was perceived by some participants as being helpful when falling asleep. Child 3 found video watching to be beneficial in the process of falling asleep: *“My mom lets me keep the laptop when I’m going to bed because then I just fall asleep watching something.”* Child 12 found the TV helpful: *“Well the TV makes my eyes tired so when I go to bed after watching it, I fall asleep straight away.”*

#### 3.8.3. Sibling Behaviour

Sibling behaviour was commonly reported as a sleep barrier. When asked about what makes it difficult to sleep, Child 3 explained that *“when my brother cries, that’s also not really helping.”* Child 5 described how she sometimes does not get enough sleep *“because of my sister. She always makes the loud noises.”* Child 6 added that their sister’s behaviour also disrupts their sleep: *“My sister, she even talks in her sleep. One night my mum was sleeping in my bed and I had to sleep in my sister’s bed and she even she kicked me off the bed in her sleep.”*

#### 3.8.4. Sleep Environment

Furthermore, the child’s sleep environment was perceived to affect sleep duration and quality. Child 3 explained that they find it difficult to go to sleep *“when the pillow is basically too hot”*. Child 4 found *“the light”* to be a barrier to sleep. Participants commonly described how a noisy sleep environment negatively affected sleep quality. Child 7 explained how neighbours’ renovations disrupted their sleep: *“Upstairs they’re doing some kind of renovation… they do it late at night and they’re literally just hammering the wall in the middle of the night and I can’t sleep with it.”* Child 6 spoke about how noise from her neighbours woke her up: *“One night I was asleep, and next thing the neighbours started shouting and woke me up.”* Child 5 explained how a neighbour’s dog prevented him from sleeping: *“The dog was barking, barking, barking and we couldn’t even sleep.”* Child 11 added that noise from the nearby road can disrupt sleep: *“The noises of the cars. Because my building, there are so many cars near, and there’s always sirens going off.”* Child 9’s sleep was disturbed by others in the neighbourhood: *“What I hate, is there’s always these gangs hanging around my house just shouting. It’s like they drank some alcohol or something. And they’re just screaming everywhere.”*

When considering perceived sleep aids, numerous participants felt that comfort items aided sleep. Child 4 said that *“hugging a pillow”* helped them to sleep because *“it’s just so comfy.”* Child 1 said that *“hugging my Squishmallow and wearing my blind fold”* helped them to sleep. Child 3 mentioned that they like to fall asleep *“holding (their) favourite Pokémon card.”*

In addition, relaxing activities at bedtime were perceived to support sleep among participants. Child 10 described how they found taking *“a shower, a warm one”* before bed helped them to sleep. Child 9 described how they *“put on some music”* which helped them to *“doze off.”* Child 9 also used their imagination to relax and fall asleep: *“I just feel like I’m on a cloud. And I imagine stars around me. And I just doze off.”* Daytime energy use was also described as a sleep aid. Child 5 explained that *“using up all my energy”* and having *“a very busy day”* makes them tired and helps them to fall asleep easily. Participants found a dark sleep environment to be a sleep aid. Child 11 said that, to help them sleep, their *“room is pitch black”*. Child 12 agreed and said that they *“like the dark”* when they are going to sleep. Child 9 also believed that *“darkness can help you sleep”.* Technology, particularly TV and video watching, was sometimes described as a sleep aid. For example, Child 6 said, *“If I watch TV so much, it helps me to sleep.”* Child 8 added, *“Me too, because it makes my eyes feel tired.”*

### 3.9. Theme 3: Daytime Functioning

This theme examines the impact of sleep on daytime functioning as perceived by the children. This theme is divided into two sub-themes: emotional and physical well-being and school performance.

#### 3.9.1. Emotional and Physical Well-Being

Participants explained that poor sleep can negatively impact their *emotional and physical well-being* throughout the day. Some children discussed how poor sleep led to mood changes. Child 1 said, “I feel really annoyed sometimes when I don’t get enough sleep. And I start to feel really, really tired as the day goes on.” Child 9 said that they feel *“very grumpy”* when they experience a late bedtime. The child described how this may impact relationships as *“you can end up hurting other people’s feelings when you’re grumpy in school”* as a result of inadequate sleep. Physical well-being was also perceived as being negatively impacted. Child 6 commented on how a lack of sleep can affect their eyes: *“your eyes go itchy if you don’t go to sleep.”* Child 12 also said that a lack of sleep has an impact on his vision: *“My eyes go blurry and then I can’t see properly.”*

#### 3.9.2. School Performance

In terms of school performance, Child 4 stated that *“I do less than what’s expected”* in school after a poor night’s sleep, while Child 11 said *“When we don’t sleep, we don’t learn properly.”* Child 5 explained how poor sleep can negatively impact concentration: *“When I have a bad night’s sleep, I can’t really concentrate because I’m very tired.”*

### 3.10. Theme 4: Technology and Parental Influence

This theme encompassed the children’s views on technology, their technology usage, and the perceived impact that technology use had on their sleep onset and sleep quality. This theme also covered how parental restrictions and behaviours influenced sleep, as perceived by the children. This theme is divided into three sub-categories: impact of technology use, device accessibility and schedules, and parental restrictions.

#### 3.10.1. Device Accessibility and Schedules

Mixed results were found regarding device accessibility and schedules. Some parents allowed their children to have devices in their sleep environment. For example, Child 7 said, *“My mom lets me keep the laptop because since I’m still a kid I have to feel like one.”* Other children discussed not having access to devices in their bedrooms. Child 5 said, *“my mum has to take any screens out of our bedroom”* due to their sibling’s device use disrupting sleep at night. Others allowed some device use in the evenings, but curtailed it at bedtime. For instance, Child 6 said, *“So my parents know that I don’t go to sleep so they put me on the TV for a while, and when it’s 9 o’clock, I go to bed.”* Other parents only allowed devices to be used in the child’s bedroom on the weekends. For example, Child 7 said they only use devices in their bedroom on Saturdays and Sundays, and when asked about using devices in their bedroom during the week, they responded with *“Nah, I’m not allowed. Monday to Thursday I’m not allowed.”*

#### 3.10.2. Parental Restrictions

Most children had bedtimes that were set by their parents. For example, when asked about their bedtime, Child 2 responded with *“My one’s 9”*. Child 4 agreed and said, *“Yeah, my one’s 9.”* Children generally did not question their bedtimes and accepted the bedtime that their parents set. Parents also generally seemed to be aware that children needed earlier bedtimes when they need to wake up earlier. For example, Child 6 explained: *“I can’t stay up on Fridays. I’ve got to be super early on Saturday mornings. I’ve got to get up at 6am to be at swimming for 8.”* Many parents restricted device usage in bedrooms at bedtime, but the restrictions were not always followed. For instance, Child 6 explained that her parent would restrict device use and encourage her and her sister to read instead at bedtime. *“My sister has a phone, and she puts it in the middle of the book. My mum’s making her read and now she’s playing some games.”*

## 4. Discussion

This study explored primary school children’s sleep from their perspective, with particular attention to the perceived influence of technology, parental practices and environmental factors. By comparing children’s views from India and the UK, the findings offer insight into how sleep perceptions are shaped by cultural context and by the interplay of family routines, technology use, sleep environments such as noise and household conditions, as well as broader social expectations.

The findings indicate that children in both countries viewed sleep as essential for boosting energy, recovery, and growth, while poor sleep was associated with irritability, physical discomfort, and challenges with motivation, concentration, and learning at school [40]. Notably, Indian children displayed a better understanding of the impact of sleep in relation to general health. They were more familiar with the recommended sleep hours per age group, referenced related health issues such as cardiovascular conditions and diabetes, and even mentioned skin conditions, such as the appearance of dark circles. This, however, does not support previous studies, which suggest that receiving sleep education can support sleep outcomes [41].

There was a notable difference in average bedtimes between the two groups: while UK children typically went to sleep between 9 and 10 p.m., Indian primary school children generally had a bedtime ranging from 11 p.m. to 1 a.m. When discussing barriers to sleep, children from both India and the UK identified psychological factors like overthinking as significant obstacles even at a primary school age. Interestingly, neither homework nor stress were mentioned as a sleep barrier in this study in UK children while Indian children pointed to stress, heavy school workloads, and demanding daily routines as factors that delay their bedtime and postponed relaxation routines while UK children placed greater emphasis on the sleep environment as a potential barrier, expressing issues like noise from nearby activities, neighbours, and pets, as well as factors such as the temperature of their bedrooms and the condition of their bedding. Indian children, however, did not mention sleep environments whatsoever. One more distinction in perceived barriers was related to sibling behaviour. UK children identified their siblings as a factor affecting their sleep, whereas Indian children did not mention their siblings at all throughout the interviews. Children in both groups reported having nightmares, which appeared to have a strong connection to their special interests in consuming horror content in TV shows and storybooks.

The current study also explored potential sleep aids as seen from the children’s perspective. Both groups largely agreed that they found comfort in their soft toys and pillows. There were some differences in preferred nighttime routines. UK children leaned towards relaxing activities such as having a warm shower and listening to music, whereas Indian children were more inclined to read or listen to bedtime stories and enjoy a hearty and late dinner. Moreover, Indian children often found solace in religious hymns to help them fall asleep. Religious recitations or prayers seemed to form a familiar and predictable part of daily life and are often incorporated into evening routines by caregivers. Repeated exposure to such practices may therefore function as a conditioned sleep cue, providing a sense of comfort, emotional security and routine at bedtime. For children, these familiar auditory practices may support relaxation and emotional regulation, thereby facilitating sleep onset. This finding highlights how culturally situated practices can shape children’s sleep behaviours indirectly through family-level routines.

The role of technology and media, however, was a topic of conflicting outcomes when discussing barriers and aids to sleep. It yielded varying opinions from children in both India and the UK. While some viewed it as a barrier to sleep, others noted that it helped them relax and fall asleep. Both groups strongly concurred that technology is a primary factor that compels them to stay up later than intended, even if it is an aid or a form of relaxation. This is in line with previous findings, which showed that 46.2% of an adolescent sample described TV watching as a sleep aid, while the remainder described this activity as a sleep barrier (e.g., [42]). Passive media consumption appeared to support relaxation and emotional comfort by creating a familiar bedtime, which they associated with easier sleep onset. Recognising this perceived benefit is valuable, as it provides insight into how children and families currently use technology within bedtime routines, which can help inform more effective and realistic sleep education strategies that acknowledge children’s experiences while supporting families to gradually shape better bedtime routines [43].

The primary differences observed were in the types of media consumed. Indian children, even at the primary school level, had access to social media platforms such as Facebook, Snapchat, Instagram and WhatsApp, often using their own devices or their parents’ devices. In contrast, children in the UK tended to gravitate more towards playing video games. In the majority, children from both groups generally agreed that their use of technology and media the night before had a noticeable impact on how well they functioned at school.

There appeared to be stricter parental restrictions on bedtime routines and technology use among UK children. Parents often instructed their children to stop using devices and go to bed or encouraged them to read a book. In contrast, while Indian parents did eventually take away their children’s devices, this usually occurred much later in the night, around the time the adults in the household decided to go to sleep. There were instances where UK children were not completely honest about putting away their devices when asked. Some found sneaky ways to continue using their phones. On the other hand, Indian children appeared to be more honest with their parents in this regard. Overall, parents in the UK appeared to have a clearer understanding of the need for earlier bedtimes, particularly when children had to wake up early for both school and weekend activities [44,45]. On the other hand, Indian parents, as perceived by their children, sometimes expected them to wake up early despite having late bedtimes or insufficient sleep.

Although the sample size was modest, responses across the focus groups began to show considerable overlap after a limited number of interviews, suggesting that thematic saturation was reached within the sampled populations. This repetition of themes across groups supports the credibility of the findings and indicates that the core shared themes were meaningful within these contexts. Nevertheless, by placing children’s perspectives at the centre of the analysis, this study contributes to an underrepresented area of sleep research and highlights the importance of incorporating children’s voices when developing sleep education initiatives and family-based interventions.

### Limitations and Recommendations

There are a few limitations present in this study. First, the sample size is quite small, with only 12 participants from the UK and 15 from India, which may not adequately represent the broader primary school-aged population. Although the focus groups produced similar responses, this small number restricts the ability to generalise the findings. Additionally, there is a concern regarding social desirability bias; participants might have provided answers they believed were more acceptable to their peers and the researcher. This may not have been the case if children were interviewed on a 1:1 basis. Since children live and interact closely with their parents, the study did not capture the parents’ perspectives on their routines. Including these viewpoints would have allowed for a more comprehensive comparison and evaluation of responses regarding routine timings and home restrictions, ultimately providing deeper insights into family dynamics across cultures.

Second, although the study aimed to explore cultural and environmental influences on sleep, the observed differences between the Indian and UK samples cannot be attributed solely to cultural contexts. Other unmeasured factors, such as parental socio-economic status, educational level, housing conditions and neighbourhood environments, may have influenced children’s sleep practices and perceptions within each setting.

Future studies could delve deeper into family sleeping patterns and benefit from employing mixed-method approaches when exploring sleep aids and barriers among children, thereby enhancing the validity of the findings. In addition to interviews, researchers might consider using more objective data collection techniques, such as autographic measures, to accurately capture sleep/wake times and overall sleep duration. It would also be advantageous to conduct similar research with diverse populations, including children from various socioeconomic and ethnic backgrounds, like the current study, but on a larger scale.

## 5. Conclusions

This study examined primary school children’s perceptions of sleep in India and the UK, focusing on children’s understanding of sleep, bedtime technology use and the influence of family routines and parental practices on sleep behaviours. By centring children’s voices, the study addresses a gap in sleep research that has largely relied on adult or clinical perspectives.

The findings show that children across both settings recognise sleep as important for daily functioning, emotional well-being and school engagement, although their understanding is frequently framed through adult-oriented health narratives rather than child-relevant experiences. Children also described technology as a central influence on sleep, reporting its role as both a barrier to sleep and, in some cases, a perceived aid to falling asleep. This contrast highlights a misalignment between children’s conceptual knowledge about sleep and their lived experiences and everyday sleep practices.

Importantly, children’s accounts indicate that sleep behaviours are closely embedded within family routines and parental expectations. While parents were perceived as setting rules around bedtimes and technology use, families may not always be fully aware of how daily schedules affect children’s sleep and next-day functioning. These findings emphasise the importance of awareness, support and education that meaningfully combine children’s voices into family routines that promote consistent, developmentally appropriate sleep practices within everyday settings. Moreover, parents should also pay close attention to the type of content children are exposed to and be mindful of their own technology use during their children’s bedtime routines.

## Data Availability

Due to ethical concerns and data protection policies, University College London (UCL) does not authorise the sharing of raw participant data beyond the university’s systems.
